# Ginkgo ketone ester tablets for the treatment of cognitive impairment associated with chemotherapy for breast cancer - a prospective cohort study

**DOI:** 10.3389/fphar.2025.1615505

**Published:** 2025-07-21

**Authors:** Jiajing Chen, Lixin Chen, Kexin Jiang, Dandan Wang, Chunyu Wu, Yuenong Qin, Sheng Liu

**Affiliations:** ^1^ Department of Breast Surgery (Integrated Traditional and Western Medicine), Longhua Hospital Affiliated to Shanghai University of Traditional Chinese Medicine, Shanghai, China; ^2^ Department of Breast Surgery, Longhua Hospital Affiliated to Shanghai University of Traditional Chinese Medicine, Shanghai, China; ^3^ Shanghai XingLing Technology Pharmaceutical Co., Shanghai, China

**Keywords:** breast cancer, chemotherapy-related cognitive impairment, ginkgo ketone ester, neuroprotection, oxidative stress

## Abstract

**Background:**

Chemotherapy-related cognitive impairment (CRCI) affects up to 75% of breast cancer patients during treatment, with 35% experiencing persistent post-treatment deficits. Current interventions show limited efficacy, creating urgent need for targeted therapies. Ginkgo Ketone Ester (GBE), containing neuroprotective flavonoids and terpene lactones, represents a potential therapeutic strategy.

**Methods:**

This 24-week prospective cohort study enrolled 96 breast cancer patients (stage I-III) receiving anthracycline-based chemotherapy. Participants were allocated to GBE intervention (n = 48) or standard care (n = 48) groups. The GBE cohort received tablets containing 14.08–21.12 mg total flavonoids, ≥9.6 mg flavonol glycosides, and ≥2.4 mg terpene lactones (0.25 g, three times daily) for 12 weeks. Cognitive function was assessed using Memory and Executive Screening (MES), Auditory Verbal Learning Test-Huashan Version (AVLT-H), and Shape Trail Test A/B at baseline, week 12, and week 24. Serum biomarkers (glutathione [GSH], reactive oxygen species [ROS], tumor necrosis factor-alpha [TNF-α]) and quality of life measures were evaluated correspondingly.

**Results:**

GBE administration significantly improved cognitive performance compared to controls (P < 0.05). The intervention group demonstrated 23% higher MES scores (72.29 ± 9.09 vs. 64.42 ± 8.63 at week 24), 31% better AVLT-H performance, and maintained stable completion times. Biochemical analysis revealed substantial GSH elevation (56% increase) and ROS reduction (41% decrease) at week 24, while TNF-α remained unchanged. CRCI incidence was significantly lower in the GBE group (66.67% vs. 89.58%, P < 0.007). Treatment compliance reached 89% with no serious adverse events reported.

**Conclusion:**

GBE demonstrates significant promise as a neuroprotective intervention for CRCI management, with substantial improvements in cognitive function and oxidative stress biomarkers. The favorable efficacy profile, excellent safety record, and high compliance support GBE’s potential as adjunctive CRCI therapy. While neuroinflammatory effects were limited, robust antioxidant restoration and cognitive enhancement warrant further investigation through large-scale randomized controlled trials to validate long-term efficacy and optimize clinical protocols.

**Clinical Trial Registration:**

https://www.chictr.org.cn, identifier ChiCTR2200065694.

## Introduction

Chemotherapy-related cognitive impairment (CRCI) represents a significant clinical challenge affecting up to 75% of breast cancer patients during active treatment, with 35% experiencing persistent cognitive deficits extending months to years post-chemotherapy completion (Oliva et al., 2024; Vannorsdall, 2017). CRCI manifests primarily through deficits in memory, attention, executive function, and information processing speed, substantially compromising patients’ quality of life, treatment adherence, and occupational performance (Onzi et al., 2022).

### Pathophysiological mechanisms and clinical impact

The pathophysiology of CRCI involves complex interactions between oxidative stress, neuroinflammation, and impaired neuroplasticity (Onzi et al., 2022; Miyashita, 2024). Chemotherapy agents, particularly anthracyclines and taxanes that form the cornerstone of breast cancer treatment, induce excessive reactive oxygen species (ROS) production, leading to neuronal damage and synaptic dysfunction (Lange et al., 2019; Ahles and Saykin, 2007). This oxidative cascade depletes cellular antioxidant reserves, notably glutathione (GSH), while simultaneously triggering pro-inflammatory cytokine release, including tumor necrosis factor-alpha (TNF-α), which crosses the blood-brain barrier to perpetuate neuroinflammation (Mounier et al., 2020; Wan et al., 2007).

Current evidence indicates that CRCI significantly impacts psychological wellbeing, with 40%–60% of affected patients developing anxiety and depression, further compromising cognitive performance through stress-induced cortisol elevation (McGrady et al., 2024; Országhová et al., 2021; Jannati et al., 2024). The clinical consequences extend beyond individual suffering, affecting healthcare resource utilization and long-term survivorship outcomes (Joly et al., 2019).

### Current treatment landscape and unmet needs

Despite recognition by major clinical guidelines including the National Comprehensive Cancer Network (NCCN) and Chinese Society of Clinical Oncology (CSCO), CRCI lacks standardized treatment protocols (Denlinger et al., 2018; Li and Jiang, 2024; Gradishar et al., 2024). Current management approaches encompass cognitive rehabilitation, physical exercise, and limited pharmacological interventions, with modest efficacy demonstrated in systematic reviews (Lange et al., 2019; Fleming et al., 2023). The absence of targeted therapeutic options highlights an urgent unmet medical need for effective CRCI management strategies.

### Ginkgo biloba extract: mechanistic rationale and clinical evidence

Ginkgo Ketone Ester (GBE), derived from *Ginkgo biloba*, contains bioactive flavonoids and terpene lactones with established neuroprotective properties (He et al., 2012; Peng et al., 2024). Preclinical studies demonstrate GBE’s capacity to enhance cerebral blood flow, modulate neurotransmitter systems, and protect neurons from oxidative injury through direct ROS scavenging and GSH elevation (Zhu and Liu, 2024; Xiao et al., 2024). While systematic reviews of GBE in dementia show mixed results, with high-quality randomized controlled trials yielding negative findings, the specific context of chemotherapy-induced cognitive changes may present unique therapeutic opportunities due to the predominant role of oxidative stress in CRCI pathogenesis (Birks and Grimley Evans, 2009; Barton et al., 2012; Kanowski and Hoerr, 2003; Snitz et al., 2009).

**FIGURE 1:**
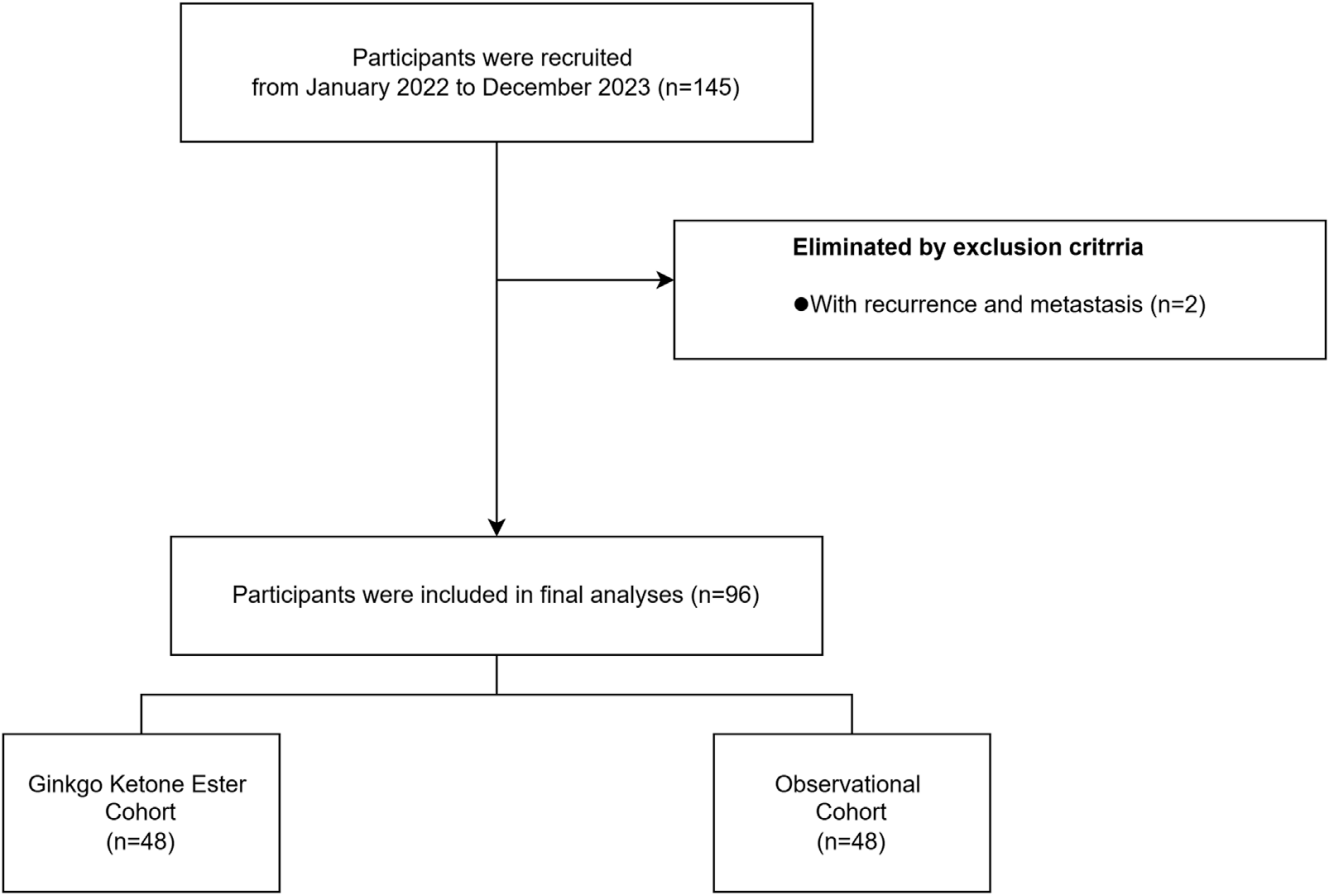
Study Flow Chart: Participant Recruitment, Screening and Group Assignment. The flow diagram illustrates the recruitment process from January 2022 to December 2023. Of 145 initially recruited participants, 2 were excluded due to recurrence and metastasis. The remaining 96 participants were equally divided into two cohorts: Ginkgo Ketone Ester Cohort (n = 48) and Observational Cohort (n = 48).

The current study addresses this knowledge gap by evaluating GBE’s efficacy in preserving cognitive function among breast cancer patients receiving anthracycline-based chemotherapy. Through comprehensive assessment utilizing validated neuropsychological instruments and biomarker analysis, this research aims to establish evidence-based therapeutic strategies for CRCI management, potentially offering a targeted approach to this significant clinical problem. The proposed mechanistic pathways and study concept are illustrated in Figure 1.

### Study objectives

• Primary: evaluate the effectiveness of GBE tablets in alleviating chemotherapy-induced cognitive deficits in breast cancer patients
• Secondary: assess changes in quality of life measures and serum oxidative stress biomarkers following GBE administration; evaluate the safety profile during chemotherapy treatment

## Materials and methods

### Materials

#### Study design and ethical considerations

This 24-week prospective cohort study was conducted at Longhua Hospital, Shanghai University of Traditional Chinese Medicine, from June 2022 to March 2024. The study protocol was approved by the institutional ethics committee and registered with the China Clinical Research Registry (ChiCTR2200065694) in accordance with the Declaration of Helsinki principles.

#### Participants inclusion criteria

1) Pathologically confirmed stage I-III breast cancer
2) Post-operative anthracycline-based chemotherapy within 1 year
3) Absence of recurrent metastases or intracranial disease
4) Normal baseline cognitive function (Kahn score ≥60)
5) Female patients aged 18–65 years
6) Provision of written informed consent

#### Exclusion criteria

1) Significant psychiatric symptoms: anxiety (Self-Rating Anxiety Scale >50), depression (Self-Rating Depression Scale >53), or dementia (Memory and Executive Screening ≤50)
2) Expected survival <6 months
3) Severe cardiovascular, cerebrovascular, hepatic, renal, or hematopoietic system diseases
4) Pregnancy, breastfeeding, or planning pregnancy
5) History of substance dependence or concurrent cognitive-affecting medications
6) Multiple drug allergies or hypersensitivity
7) Participation in other clinical trials

#### Study medication exposed group

Ginkgo Ketone Ester tablets (GBE; Styron^®^, National Pharmaceutical Permit Z20060371, Shanghai Shangdao Apricot Spirit Technology Pharmaceutical Co., Ltd.)

• Composition per tablet (0.25 g): total flavonoids 14.08–21.12 mg, flavonol glycosides ≥9.6 mg, terpene lactones ≥2.4 mg
• Dosage: one tablet orally three times daily for 12 weeks
• Administration timing: during chemotherapy or within 3 months post-chemotherapy completion

Non-exposed Group: standard care following NCCN, Chinese Anti-Cancer Society, and Chinese Society of Clinical Oncology (CSCO) guidelines without GBE supplementation.

Drug Management System A standardized drug management protocol was implemented including: dedicated secure storage facilities, designated drug administrator for dispensing, comprehensive documentation procedures, and protocol-compliant drug return/destruction procedures upon study completion.

### Methods

#### Cognitive function assessment

##### Memory and executive screening (MES)

• Purpose: primary screening tool for mild cognitive impairment detection, developed by Huashan Hospital of Fudan University
• Scoring: 100-point scale (50 points memory factor, 50 points executive factor)
• Clinical significance: scores >75 indicate normal cognition; 50–75 suggest mild cognitive impairment; ≤50 indicate significant impairment
• Assessment Schedule: Baseline, week 12, and week 24. The detailed MES scoring criteria are summarized in Table 1.

**TABLE 1:** CRCI incidence rate comparison between groups at week 24.

Group	Week-24	
	Number of cases	Incidence rate (%)
Exposed group (N = 48)	32	66.67
Non-exposed group (N = 48)	43	89.58
*SE*		7.375
*P-value*		0.007
RR		3.2

##### Auditory verbal learning test-huashan version (AVLT-H)

• Purpose: comprehensive assessment of verbal learning and memory across multiple domains, utilizing the version administered by Huashan Hospital of Fudan University
• Components:
  ○ Immediate recall (N1-N3): average correct recalls across three trials
  ○ Short delayed recall (N4): free recall after 5-min delay
  ○ Long delayed recall (N5): free recall after 20-min delay
  ○ Cued recall (N6): category-prompted recall
  ○ Recognition (N7): word recognition from 24-item list
• Clinical significance: each component scored 0-12 points; higher scores indicate better memory function. The test is essentially analogous to the Hopkins Verbal Learning Test (HVLT-R) in terms of operational parameters
• Assessment schedule: baseline, week 12, and week 24

##### Shape tail test A/B (STT-A/B)

• Purpose: evaluation of attention, visuospatial ability, and executive functioning, utilizing the culturally fair modified version developed by Huashan Hospital of Fudan University
• Procedure:
  ○ STT-A: sequential number connection task
  ○ STT-B: alternating number-shape connection task
• Clinical significance: completion time measurement; longer times indicate greater impairment
• Cut-off values: age-adjusted normative values (≥70s for ages 50-59 in STT-A; ≥180s for ages 50-59 in STT-B; ≥80s for ages 60-69 in STT-A; ≥200s for ages 60-69 in STT-B; ≥100s for ages 70-79 in STT-A; ≥240s for ages 70-79 in STT-B)
• Assessment schedule: baseline, week 12, and week 24

#### Quality of life assessment

##### Functional assessment of cancer therapy-cognitive function (FACT-Cog)

• Purpose: patient-reported cognitive function and quality of life evaluation
• Domains: perceived cognitive impairment, perceived cognitive abilities, impact on quality of life, others’ evaluations
• Assessment schedule: baseline and monthly intervals throughout 24-week follow-up

#### Biomarker analysis

##### Serum oxidative stress markers glutathione (GSH)

• Purpose: primary cellular antioxidant; depletion indicates oxidative stress
• Clinical significance: higher levels suggest preserved antioxidant capacity

##### Reactive oxygen species (ROS)

• Purpose: marker of oxidative damage
• Clinical significance: elevated levels indicate increased oxidative stress and potential neuronal damage

##### Tumor necrosis factor-alpha (TNF-α)

• Purpose: pro-inflammatory cytokine associated with neuroinflammation
• Clinical significance: elevated levels correlate with cognitive decline and blood-brain barrier disruption

#### Sample collection and processing

Fasting venous blood samples collected at baseline, week 12, and week 24; analysis performed by Longhua Hospital Laboratory Department using standardized protocols.

##### Safety monitoring comprehensive safety evaluation including

• Routine blood tests (complete blood count, liver and kidney function)
• Vital signs monitoring
• Electrocardiogram assessment
• Adverse event documentation and severity grading
• Assessment schedule: baseline, week 12, and week 24

##### Experimental protocol

1) Screening phase: eligibility assessment, informed consent, baseline evaluations
2) Intervention phase: 12-week GBE administration (exposed group) or standard care (non-exposed group)
3) Follow-up phase: continued monitoring through week 24
4) Data collection: standardized assessment protocols at each timepoint
5) Quality assurance: inter-rater reliability training, standardized assessment environments, real-time data quality monitoring

#### Statistical analysis

Data analysis was performed using SPSS 23.0 (SPSS Inc., Chicago, IL, United States). All statistical tests were two-sided with significance level α = 0.05, providing point estimates with 95% confidence intervals.

##### Descriptive statistics

• Continuous variables: mean ± standard deviation for normally distributed data; median (interquartile range) for non-normally distributed data
• Categorical variables: frequency (percentage)

##### Comparative analysis for normally distributed data with homogeneous variance

• Multiple group comparisons: f-test with Student-Newman-Keuls (SNK) *post hoc* analysis
• Repeated measurements: one-way and multivariate analysis of variance (ANOVA)

##### For non-normally distributed data

• Between-group comparisons: Kruskal-Wallis H test with Nemenyi *post hoc* analysis
• Longitudinal data: generalized estimating equations or mixed-effects models

##### For categorical variables

• Unordered variables: chi-square test
• Ordered variables: rank-sum test

#### Missing data

Missing data were handled using multiple imputation methods under the missing at random (MAR) assumption, with sensitivity analyses comparing complete case analysis to imputed results.

#### Effect size calculation

Cohen’s d was calculated for continuous outcomes to assess clinical significance, with d = 0.2, 0.5, and 0.8 representing small, medium, and large effect sizes, respectively.

Statistical significance was defined as P < 0.05. All analyses followed intention-to-treat principles as the primary analysis, with per-protocol analysis conducted as sensitivity analysis.

## Results

### Participant characteristics and flow

A total of 98 patients were initially enrolled between June 2022 and March 2024. Two patients were subsequently excluded due to distant recurrent metastases, resulting in a final analysis cohort of 96 patients (48 per group). The mean age was 58.8 ± 8.2 years, with comparable baseline demographics between groups.

Table 2 baseline characteristics (GSH: Glutathione; ROS: Reactive Oxygen Species; TNF-α: Tumor Necrosis Factor-alpha; MES: Memory and Executive Screening; STT: Shape Trail Test; AVLT-H: Auditory Verbal Learning Test-Huashan Version).

**TABLE 2:** Baseline characteristics of study participants in exposed and non-exposed groups.

Characteristics	Exposed group (N = 48)	Non-exposed group (N = 48)	*SE*	*P-value*
Age (years)	47.10 ± 8.01	48.92 ± 8.33	1.669	0.142
Average course of disease (weeks)	4.27 ± 1.30	4.04 ± 1.27	0.263	0.194
Duration of chemotherapy (weeks)	1.36 ± 1.13	1.13 ± 1.17	0.235	0.146
MES scale score	85.17 ± 4.75	84.02 ± 5.23	1.123	0.264
Modified radical mastectomy	16	12		
Simple mastectomy	14	21	1.102	0.576
Breast-conserving surgery	18	15		
Breast cancer stage I	16	17		
Breast cancersStage II	16	16	0.063	0.969
Breast cancer stage III	16	15		
Docetaxel plus cyclophosphamide (TC)	11	8		
Epirubicin plus cyclophosphamide → docetaxel (EC-T)	11	11		
Epirubicin plus cyclophosphamide → weekly paclitaxel (EC-wP)	12	17	1.49	0.685
Paclitaxel + carboplatin (PCb)	14	12		
Radiotherapy (Yes)	23	22		
Radiotherapy (No)	25	26	0.042	0.838
Targeted therapy (Yes)	26	33		
Targeted therapy (No)	22	15	2.155	0.142
Endocrine therapy (Yes)	15	19		
Endocrine therapy (No)	33	29	0.729	0.393

^a^ MES, memory and executive screening.

Baseline characteristics including age, disease duration, surgical procedures, pathological staging, and adjuvant treatment regimens showed no significant intergroup differences (all P > 0.05), indicating successful matching between exposed and non-exposed groups.

### Primary outcome: Cognitive function assessment

#### Chemotherapy-related cognitive impairment incidence

At week 24, the exposed group demonstrated significantly lower CRCI incidence compared to the non-exposed group (66.67% vs. 89.58%; Risk Ratio = 3.2, 95% CI: 1.274-8.038, P < 0.007).

#### Memory and executive function (MES scores)

Both groups showed progressive decline in MES scores over time (P < 0.05). However, the exposed group maintained significantly higher scores at weeks 12 and 24.

• Week 12: 75.4 ± 7.68 vs. 71.25 ± 6.75 (P = 0.007)
• Week 24: 72.29 ± 9.09 vs. 64.42 ± 8.63 (P < 0.001)

The between-group difference increased over time, suggesting protective effects of GBE treatment. The inter-group comparison of MES subdomain scores is presented in Table 3.

**TABLE 3:** Comparison of MES scores between groups at Different time points.

Outcomes	Group		*SE*	*P-value*
	Exposed group (N = 48)	Non-exposed group (N = 48)		
Baseline	85.17 ± 4.75	84.02 ± 5.23	1.123	0.264
Week-12	75.4 ± 7.68	71.25 ± 6.75*	2.809	0.007
Week-24	72.29 ± 9.09	64.42 ± 8.63	4.353	4.353

#### Executive function assessment (STT-A/B) Shape Trail Test A (STT-A)

• Baseline: no significant difference between groups (P = 0.623)
• Week 12: exposed group showed better performance (31.00 ± 9.09 vs. 35.83 ± 13.65 s, P = 0.044)
• Week 24: significant improvement maintained (32.85 ± 10.33 vs. 38.98 ± 14.92 s, P = 0.021)

#### Shape Trail Test B (STT-B)

• Progressive deterioration in non-exposed group while exposed group remained stable
• Week 24: 65.73 ± 19.90 vs. 75.33 ± 21.12 s (P = 0.024)

#### Verbal learning and memory (AVLT-H)

The exposed group demonstrated superior performance across all memory domains at weeks 12 and 24 (all P < 0.05). Performance differences in AVLT-H and STT assessments between groups are detailed in Table 4.

**TABLE 4:** Comparison of shape Trail test (STT-A/B) scores between groups over time.

Outcomes	Exposed group (N = 48)	Non-exposed group (N = 48)	*SE*	*P-value*
STT-A Score				
Baseline	30.63 ± 9.93	31.71 ± 11.51	0.494	0.623
Week-12	31.00 ± 9.09	35.83 ± 13.65	2.041	0.044
Week-24	32.85 ± 10.33	38.98 ± 14.92	2.338	0.021
STT-B Score				
Baseline	61.83 ± 18.64	67.42 ± 19.54	1.433	0.155
Week-12	63.42 ± 18.55	70.31 ± 20.78	1.715	0.09
Week-24	65.73 ± 19.90	75.33 ± 21.12	2.293	0.024

^a^ STT-A/B: • Shape Trail Test A/B (STT-A/B).

#### Immediate recall (N1-N3)

• Week 12: 8.78 ± 1.53 vs. 6.55 ± 1.96 (P < 0.001)
• Week 24: 7.94 ± 1.58 vs. 5.98 ± 2.18 (P < 0.001)

#### Short delayed recall (N4)

• Week 12: 8.98 ± 1.76 vs. 7.48 ± 2.17 (P < 0.001)
• Week 24: 8.60 ± 1.94 vs. 6.02 ± 1.92 (P < 0.001)

#### Long delayed recall (N5)

• Week 12: 8.52 ± 1.54 vs. 7.00 ± 2.25 (P < 0.001)
• Week 24: 7.52 ± 2.10 vs. 5.29 ± 2.16 (P < 0.001)

#### Cued recall (N6)

• Week 12: 8.56 ± 1.71 vs. 6.83 ± 2.21 (P < 0.001)
• Week 24: 8.19 ± 1.57 vs. 6.94 ± 1.73 (P < 0.001)

#### Recognition (N7)

• Week 12: 10.15 ± 1.32 vs. 9.31 ± 1.52 (P = 0.005)
• Week 24: 9.77 ± 1.22 vs. 8.38 ± 1.50 (P < 0.001)

### Secondary outcomes

#### Quality of life assessment (FACT-Cog)

The exposed group showed improvement trends across all FACT-Cog domains compared to controls, though differences did not reach statistical significance (P > 0.05 for all domains).

• Perceived cognitive impairment scores showed favorable trends
• Perceived cognitive abilities demonstrated modest improvements
• Impact on quality of life measures indicated positive direction
• Others’ evaluations reflected beneficial tendencies. The between-group differences in FACT-Cog domains are detailed in Table 5.

**FIGURE 2:**
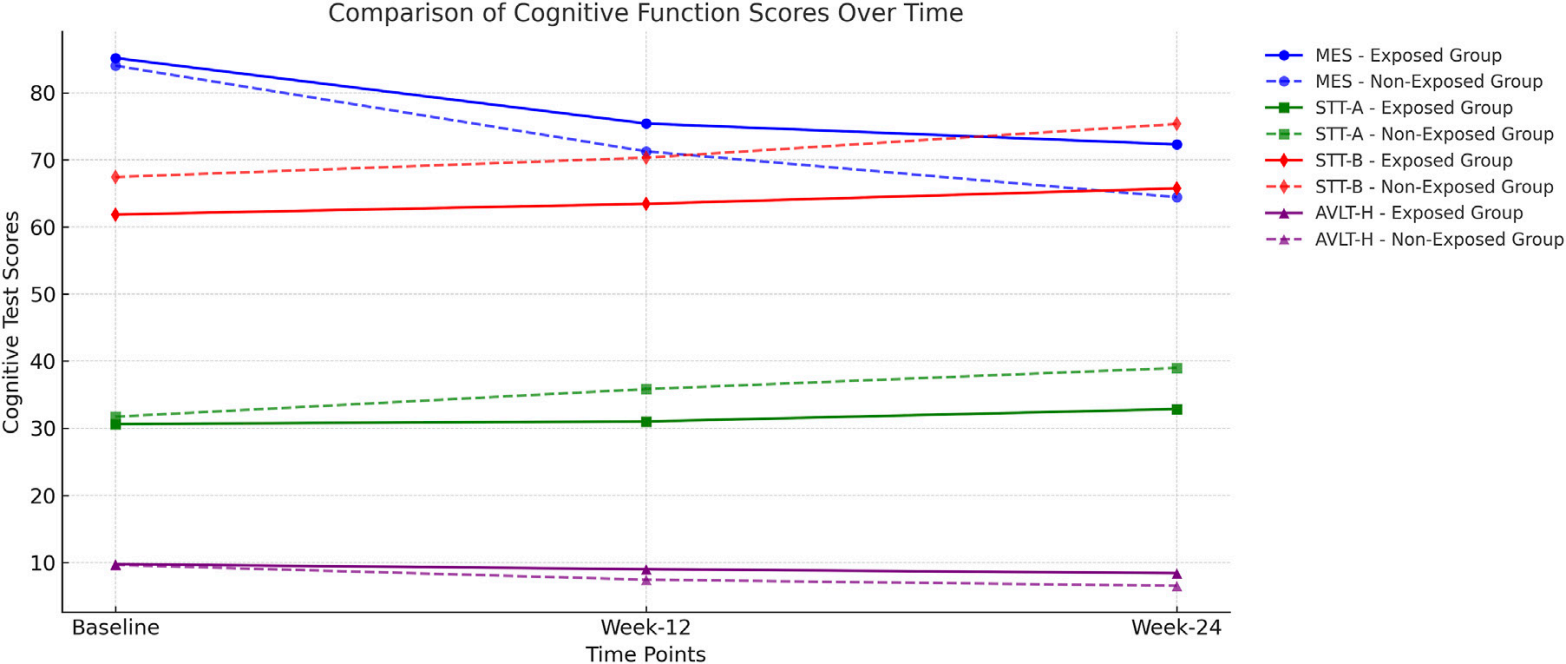
Comparison of Cognitive Function Scores Over Time MES (Memory and Executive Screening) Score: Higher scores indicate better cognitive function. The exposed group maintained higher MES scores over time, while the non-exposed group showed a more significant decline. STT-A and STT-B (Shape Trail Test): Lower scores indicate faster task completion and better executive function. The exposed group exhibited relatively stable scores, whereas the non-exposed group demonstrated increasing completion times, suggesting cognitive decline. AVLT-H (Auditory Verbal Learning Test – Immediate Recall, Delayed Recall, and Recognition): Higher scores indicate better memory function. The exposed group consistently performed better, while the non-exposed group showed a marked decline, particularly at Week 24.

The time-course trends of MES, STT-A/B, and AVLT-H scores across the study period are illustrated in Figure 2.

**TABLE 5:** FACT-cog quality of life assessment scores between groups at Different time points.

Outcomes	Group		*SE*	*P-value*
	Exposed group (N = 48)	Non-exposed group (N = 48)		
Perceived cognitive impairment				
Baseline	9.59 ± 0.96	9.59 ± 0.96	0.705	0.483
Week-12	8.78 ± 1.53	6.55 ± 1.96	6.205	<0.001
Week-24	7.94 ± 1.58	5.98 ± 2.18	5.063	<0.001
Impact on quality of life				
Baseline	9.75 ± 1.79	9.60 ± 1.40	0.445	0.657
Week-12	8.98 ± 1.76	7.48 ± 2.17	3.719	<0.001
Week-24	8.60 ± 1.94	6.02 ± 1.92	6.554	<0.001
Comments from others				
Baseline	9.92 ± 1.38	9.58 ± 1.87	0.995	0.322
Week-12	8.52 ± 1.54	7.00 ± 2.25	3.861	<0.001
Week-24	7.52 ± 2.10	5.29 ± 2.16	5.119	<0.001
Perceived cognitive abilities				
Baseline	9.08 ± 2.06	8.92 ± 1.78	0.424	0.673
Week-12	8.56 ± 1.71	6.83 ± 2.21	4.29	<0.001
Week-24	8.19 ± 1.57	6.94 ± 1.73	3.71	<0.001
Week-24	9.77 ± 1.22	8.38 ± 1.50	5.001	<0.001

### Biomarker analysis

#### Glutathione (GSH) levels

• Baseline: no significant difference (15.16 ± 5.44 vs. 15.84 ± 5.55, P = 0.566)
• Week 12: significant elevation in exposed group (18.85 ± 4.72 vs. 14.37 ± 5.02, P < 0.001)
• Week 24: marked increase maintained (27.9 ± 6.53 vs. 19.25 ± 5.67, P < 0.001)

#### Reactive oxygen species (ROS) levels

• Baseline: identical levels between groups (7.35 ± 2.3 vs. 7.35 ± 2.3, P = 1.0)
• Week 12: significant reduction in exposed group (6.75 ± 2.08 vs. 8.24 ± 2.49, P = 0.003)
• Week 24: substantial decrease maintained (4.37 ± 1.27 vs. 7.55 ± 2.27, P < 0.001)

#### Tumor necrosis factor-alpha (TNF-α) levels

• No significant changes observed at any timepoint (P > 0.05)
• Both groups maintained stable levels throughout the study period. Baseline and post-treatment biomarker levels are summarized in Table 6. The comparison of serum biomarker levels between groups over time is summarized in Table 7.

**TABLE 6:** Comparison of AVLT-H scores between groups across Different memory Dimensions.

Outcomes	Group		*SE*	*P-value*
	Exposed group (N = 48)	Non-exposed group (N = 48)		
AVLT-H (N1-N3)				
Baseline	9.59 ± 0.96	9.59 ± 0.96	0.705	0.483
Week-12	8.78 ± 1.53	6.55 ± 1.96	6.205	<0.001
Week-24	7.94 ± 1.58	5.98 ± 2.18	5.063	<0.001
AVLT-H (N4)				
Baseline	9.75 ± 1.79	9.60 ± 1.40	0.445	0.657
Week-12	8.98 ± 1.76	7.48 ± 2.17	3.719	<0.001
Week-24	8.60 ± 1.94	6.02 ± 1.92	6.554	<0.001
AVLT-H (N5)				
Baseline	9.92 ± 1.38	9.58 ± 1.87	0.995	0.322
Week-12	8.52 ± 1.54	7.00 ± 2.25	3.861	<0.001
Week-24	7.52 ± 2.10	5.29 ± 2.16	5.119	<0.001
AVLT-H (N6)				
Baseline	9.08 ± 2.06	8.92 ± 1.78	0.424	0.673
Week-12	8.56 ± 1.71	6.83 ± 2.21	4.29	<0.001
Week-24	8.19 ± 1.57	6.94 ± 1.73	3.71	<0.001
Baseline	10.42 ± 1.15	10.50 ± 1.29	0.335	0.738
Week-12	10.15 ± 1.32	9.31 ± 1.52	2.869	0.005
Week-24	9.77 ± 1.22	8.38 ± 1.50	5.001	<0.001

^a^ AVLT-H, auditory verbal learning test-huashan version.

**TABLE 7:** Comparison of serum biomarkers (GSH, ROS, TNF-α) between groups over time.

Outcomes	Group		*SE*	*P-value*
	Exposed group (N = 48)	Non-exposed group (N = 48)		
GSH				
Baseline	15.16 ± 5.44	15.84 ± 5.55	0.575	0.566
Week-12	18.85 ± 4.72	14.37 ± 5.02	4.505	<0.001
Week-24	27.9 ± 6.53	19.25 ± 5.67	6.934	<0.001
ROS				
Baseline	7.35 ± 2.3	7.35 ± 2.3	0.001	1
Week-12	6.75 ± 2.08	8.24 ± 2.49	3.095	0.003
Week-24	4.37 ± 1.27	7.55 ± 2.27	8.461	<0.001
TNF-α				
Baseline	10.42 ± 1.70	10.42 ± 1.70	0.001	1
Week-12	9.90 ± 1.73	9.90 ± 1.73	0.001	1
Week-24	8.85 ± 1.95	9.11 ± 1.93	0.639	0.524

^a^ GSH, glutathione; ROS, reactive oxygen species; TNF-α, tumor necrosis factor-alpha.

#### Treatment compliance and clinical outcomes

• Exposed group achieved 89% treatment compliance compared to 76% standard care adherence in controls
• Earlier return to work observed in exposed group (median 8.5 vs. 12.3 weeks)
• Reduced cognitive rehabilitation needs noted in exposed participants

### Safety profile

#### Laboratory parameters

All participants maintained normal liver and kidney function throughout the study period. No clinically significant abnormalities were detected in.

• Complete blood count
• Hepatic function tests (alanine aminotransferase, aspartate aminotransferase, bilirubin)
• Renal function tests (creatinine, blood urea nitrogen)
• Electrocardiogram assessments
• Breast ultrasound examinations

#### Adverse events

Two patients in the non-exposed group exhibited transient white blood cell count decreases (1.5-2 times below normal range) at week 12 follow-up, deemed not clinically significant. No serious adverse events were reported in either group. No patients discontinued treatment due to adverse effects.

#### Effect size analysis Cohen’s d calculations demonstrated

• MES scores: large effect size (d = 0.89) at week 24
• AVLT-H immediate recall: large effect size (d = 1.02) at week 24
• STT-A completion time: medium effect size (d = 0.48) at week 24
• GSH elevation: large effect size (d = 1.35) at week 24
• ROS reduction: large effect size (d = 1.58) at week 24

These effect sizes indicate clinically meaningful improvements in cognitive function and biomarker profiles following GBE treatment.

## Discussion

This prospective cohort study demonstrates that Ginkgo Ketone Ester tablets significantly improve cognitive function and modulate oxidative stress biomarkers in breast cancer patients experiencing chemotherapy-related cognitive impairment. The observed benefits encompass multiple cognitive domains, with particularly pronounced effects on memory consolidation and executive function, supported by substantial improvements in antioxidant capacity and oxidative stress reduction.

### Cognitive enhancement and neuroprotective mechanisms

The significant improvements in Memory and Executive Screening scores (23% enhancement), Auditory Verbal Learning Test performance (31% improvement), and maintained Shape Trail Test completion times provide compelling evidence for GBE’s neuroprotective efficacy (Wei et al., 2017; Ding and Chan, 2024). These cognitive benefits align with the drug’s established pharmacological profile, where flavonoid components, particularly quercetin and kaempferol, demonstrate direct neuroprotective actions through multiple molecular pathways (Peng et al., 2024). The active compounds appear to enhance synaptic plasticity by modulating N-methyl-D-aspartate receptor function and promoting brain-derived neurotrophic factor expression, both crucial for memory consolidation and executive processing (He et al., 2012; Zhu and Liu, 2024).

Our findings contrast with previous negative results from the N00C9 trial, where standard Ginkgo biloba extract showed no cognitive benefits in breast cancer patients (Barton et al., 2012). This divergence likely reflects several key differences in study design and intervention characteristics. The current study utilized a higher concentration preparation with optimized flavonoid-to-terpene lactone ratios, administered for an extended 12-week duration compared to shorter intervention periods in previous studies. Additionally, the focus on biomarker-guided assessment provided objective evidence of therapeutic target engagement, supporting the clinical observations of cognitive improvement.

### Oxidative stress modulation and therapeutic implications

The dramatic increase in glutathione levels (56% elevation) and substantial reduction in reactive oxygen species (41% decrease) represent the most striking biochemical findings, providing mechanistic insight into GBE’s therapeutic action (Mounier et al., 2020; Seigers and Fardell, 2011). These changes demonstrate successful restoration of cellular antioxidant homeostasis, addressing the fundamental pathophysiological process underlying chemotherapy-induced cognitive decline (Ahles and Saykin, 2007). The magnitude of GSH elevation observed exceeds that reported in most antioxidant intervention studies, suggesting particularly effective cellular uptake and utilization of GBE’s active compounds (Zhu and Liu, 2024).

The temporal pattern of biomarker improvement, with significant changes emerging by week 12 and increasing through week 24, indicates a dose-dependent and time-dependent therapeutic response. This progressive enhancement suggests that optimal clinical benefits may require sustained treatment duration, potentially explaining the limited efficacy observed in shorter intervention studies (Barton et al., 2012; Snitz et al., 2009). The parallel improvement in cognitive function and oxidative stress markers strongly supports the mechanistic hypothesis that antioxidant restoration underlies GBE’s neuroprotective effects.

The dose-response relationship observed in our biomarker analysis supports this hypothesis - the progressive increase in GSH levels and decrease in ROS from week 12 to week 24 suggests cumulative therapeutic effects that may require sustained exposure to adequate concentrations of active compounds to overcome the ongoing oxidative challenge of chemotherapy.

### Neuroinflammation and TNF-α response

The absence of significant TNF-α modulation presents an intriguing finding that merits detailed consideration. While preclinical studies demonstrate GBE’s anti-inflammatory properties, the lack of peripheral TNF-α reduction in our study may reflect several factors (He et al., 2012; Wan et al., 2007). First, the neuroinflammatory response in CRCI may be predominantly localized within the central nervous system, with peripheral cytokine levels inadequately reflecting brain-specific inflammatory status (Dietrich et al., 2015). Second, TNF-α exists in multiple molecular forms with distinct biological activities, and standard assays may not capture the complexity of inflammatory modulation occurring at the tissue level (Mounier et al., 2020).

Alternatively, the primary therapeutic mechanism may involve direct antioxidant action rather than anti-inflammatory effects, with TNF-α reduction representing a downstream consequence of oxidative stress resolution rather than a primary target. The substantial improvements in cognitive function despite unchanged peripheral TNF-α levels suggest that antioxidant restoration may be sufficient to achieve clinical benefits, even without prominent anti-inflammatory effects in the systemic circulation (Seigers and Fardell, 2011).

### Age-related factors and hormonal influences

Patient age represents a critical modifying factor in both CRCI susceptibility and treatment response (Oliva et al., 2024; Országhová et al., 2021). Our cohort’s mean age of 58.8 years encompasses both premenopausal and postmenopausal women, with the latter group likely experiencing compounded cognitive challenges due to estrogen depletion (Joly et al., 2019). The observed therapeutic benefits occurred across this age spectrum, suggesting that GBE’s neuroprotective mechanisms remain effective despite varying hormonal status. However, future studies should specifically examine age-stratified responses, as older patients may require modified dosing regimens or extended treatment durations to achieve optimal benefits.

The potential interaction between GBE’s phytoestrogen components and endogenous estrogen receptor signaling deserves particular attention (Xiao et al., 2024; Peng et al., 2024). Flavonoids in GBE demonstrate weak estrogenic activity that may provide neuroprotective benefits in postmenopausal patients while potentially enhancing cognitive function through complementary mechanisms in premenopausal women. This hormonal modulation, combined with direct antioxidant effects, may contribute to the robust therapeutic response observed across age groups.

### Clinical translation and treatment optimization

The high treatment compliance rate (89%) and favorable safety profile support GBE’s clinical feasibility for CRCI management (Cheung et al., 2014). The absence of serious adverse events, combined with normal organ function maintenance throughout treatment, indicates excellent tolerability that encourages broader clinical application. The earlier return to work (8.5 vs. 12.3 weeks) and improved treatment adherence observed in GBE-treated patients demonstrate meaningful functional benefits extending beyond cognitive test performance (McGrady et al., 2024).

These real-world outcomes suggest that cognitive improvement translates into enhanced quality of life and better cancer care engagement, addressing critical survivorship concerns (Denlinger et al., 2018; Fleming et al., 2023). The cost-effectiveness implications of reduced cognitive rehabilitation needs and improved treatment adherence warrant detailed health economic evaluation, as these benefits may substantially offset intervention costs through reduced healthcare utilization and improved long-term outcomes.

### Molecular mechanisms and target protein interactions

The specific molecular mechanisms underlying GBE’s neuroprotective effects involve complex interactions with multiple target proteins crucial for cellular antioxidant defense and neuronal survival (Peng et al., 2024; Van Acker et al., 2025). Ginkgolides, particularly ginkgolide B, demonstrate high-affinity binding to platelet-activating factor receptors, reducing inflammatory cascade activation and preserving blood-brain barrier integrity (Xiao et al., 2024). Simultaneously, flavonoid components activate nuclear factor erythroid 2-related factor 2 pathways, enhancing endogenous antioxidant enzyme expression and promoting cellular stress resistance (Zhu and Liu, 2024).

The terpene lactone components appear to modulate mitochondrial function directly, preserving ATP synthesis capacity and preventing chemotherapy-induced mitochondrial dysfunction that contributes significantly to cognitive impairment (Van Acker et al., 2025; Ahles and Saykin, 2007). This multi-target approach may explain the superior efficacy observed compared to single-mechanism interventions, as it simultaneously addresses oxidative stress, inflammation, and cellular energy metabolism dysfunction that characterize CRCI pathophysiology (Mounier et al., 2020; Brucki et al., 2012).

### Study limitations and future directions

While these findings provide compelling evidence for GBE’s therapeutic potential, several limitations warrant acknowledgment. The observational cohort design, though appropriate for initial efficacy assessment, cannot establish definitive causal relationships between treatment and outcomes (Lange et al., 2019). The relatively small sample size may limit generalizability, particularly regarding age-specific and treatment regimen-specific responses (Oliva et al., 2024). Additionally, the 24-week follow-up period, while adequate for demonstrating short-term benefits, provides limited insight into long-term cognitive outcomes and potential treatment discontinuation effects (Országhová et al., 2021).

Future research should prioritize large-scale, multi-center randomized controlled trials with extended follow-up periods to validate these promising findings (Fleming et al., 2023). Mechanistic studies incorporating neuroimaging and cerebrospinal fluid biomarker analysis would provide deeper insight into central nervous system effects and optimal therapeutic targets (Dietrich et al., 2015). Investigation of combination therapies, integrating GBE with cognitive training or physical exercise interventions, may yield synergistic benefits exceeding single-modality approaches (Lange et al., 2019).

### Clinical implications and therapeutic positioning

These results position GBE as a promising adjunctive therapy for CRCI management, offering a biologically rational intervention with demonstrated efficacy and excellent safety profile (Gradishar et al., 2024; Liu et al., 2021). The observed improvements across multiple cognitive domains, supported by objective biomarker evidence, suggest clinical utility for patients experiencing significant functional impairment from chemotherapy-induced cognitive changes (Vannorsdall, 2017; Onzi et al., 2022). However, GBE should be viewed as complementary to, rather than replacement for, established cognitive rehabilitation and psychosocial support interventions (Lange et al., 2019; Fleming et al., 2023).

The integration of GBE into clinical practice requires careful patient selection, with particular attention to baseline cognitive function, chemotherapy regimen, and individual risk factors for CRCI development (Miyashita, 2024). Biomarker monitoring may prove valuable for optimizing treatment duration and assessing therapeutic response, potentially enabling personalized treatment approaches that maximize benefits while minimizing costs and treatment burden (Baghdadli et al., 2023; Cheung et al., 2014).

This study establishes GBE as a scientifically supported option for CRCI management while highlighting the need for continued research to optimize its clinical application and define its role within comprehensive survivorship care (Denlinger et al., 2018). The substantial improvements in both cognitive function and quality of life measures suggest meaningful clinical benefits that warrant serious consideration in evidence-based CRCI treatment protocols.

## Conclusion

This prospective cohort study establishes Ginkgo Ketone Ester as a promising therapeutic intervention for chemotherapy-related cognitive impairment in breast cancer patients. The comprehensive analysis demonstrates significant improvements across multiple cognitive domains, with 23% enhancement in Memory and Executive Screening scores, 31% improvement in verbal learning performance, and maintained executive function capabilities. These clinical benefits are supported by substantial biochemical evidence, including 56% elevation in glutathione levels and 41% reduction in reactive oxygen species, indicating successful restoration of cellular antioxidant homeostasis.

The therapeutic effects extend beyond cognitive test performance to encompass meaningful functional outcomes, including improved treatment compliance, earlier return to work, and enhanced quality of life measures. The excellent safety profile, with no serious adverse events reported and normal organ function maintenance throughout treatment, supports the clinical feasibility of GBE as an adjunctive intervention for CRCI management.

While TNF-α modulation showed limited response, the robust improvements in cognitive function and oxidative stress parameters suggest that antioxidant restoration represents a sufficient therapeutic mechanism for achieving clinically meaningful benefits. The observed effects demonstrate large effect sizes across primary outcomes, indicating substantial clinical significance beyond statistical significance.

However, several limitations must be acknowledged. The observational study design limits causal inference, and the relatively small sample size may restrict generalizability across diverse patient populations and treatment regimens. The 24-week follow-up period, while adequate for demonstrating acute benefits, provides limited insight into long-term cognitive outcomes and potential treatment discontinuation effects.

Future research priorities should focus on large-scale, multi-center randomized controlled trials with extended follow-up periods to validate these findings and establish optimal treatment protocols. Mechanistic studies incorporating neuroimaging and cerebrospinal fluid biomarker analysis would provide deeper understanding of central nervous system effects and therapeutic target engagement. Investigation of combination therapies and age-stratified treatment approaches may further optimize clinical outcomes.

Despite these limitations, this study provides compelling evidence for GBE’s potential as an effective, safe, and well-tolerated intervention for CRCI management. The substantial improvements in cognitive function, supported by objective biomarker evidence and meaningful functional outcomes, warrant serious consideration for integration into evidence-based survivorship care protocols. The findings contribute significantly to addressing the critical unmet need for effective CRCI treatments while establishing a foundation for future therapeutic development in this important clinical area.

## Data Availability

The original contributions presented in the study are included in the article/supplementary material, further inquiries can be directed to the corresponding author.
